# Human and animal fasciolosis: Coprological survey in Narok, Baringo and Kisumu counties, Kenya

**DOI:** 10.4102/ojvr.v89i1.1954

**Published:** 2022-01-31

**Authors:** Cornelius K. Kipyegen, Charles I. Muleke, Elick O. Otachi

**Affiliations:** 1 Department of Biological Sciences, Faculty of Science, Egerton University, Nakuru, Kenya; 2 Department of Veterinary Pathology, Microbiology and Parasitology, Faculty of Veterinary Medicine and Surgery, Egerton University, Nakuru, Kenya

**Keywords:** fasciolosis, prevalence, human, domestic animals, zoonosis, Kenya

## Abstract

Fasciolosis is caused by digenean trematodes of the genus *Fasciola.* The principal definitive hosts are cattle, sheep and goats. Humans are infected as accidental hosts. Fasciolosis is one of the major neglected tropical diseases and is considered an emerging zoonotic infection. This study was aimed at determining the prevalence of human and domestic animal fasciolosis in selected counties in Kenya. Stool samples for *Fasciola* diagnosis were collected from humans and domestic animals and transported to the laboratory at Egerton University and processed using sedimentation technique and examined for the presence of eggs. A total of 272 human samples collected were all negative for *Fasciola* eggs. A total of 582 domestic animals (cattle [46.0%], sheep [29.9%] and goats [24.1%]) samples collected had overall prevalence of 30.9% for *Fasciola* infection. There was no significant differences (*p* > 0.05) between the prevalence of fasciolosis and origin of the animals, sex and season. There was a significant difference (*p* < 0.05) between the prevalence of fasciolosis and domestic animals, age and body condition. The prevalence of fasciolosis was high in two irrigation schemes which favour the breeding of intermediate host snail and grazing of animals along the irrigation canals where metacercaria of *Fasciola* parasites could be present on the vegetation. Although human fasciolosis was not detected in this study, the presence of animal fasciolosis can pose public health risk because of its zoonotic nature. Therefore, it is important to introduce measures which would help to reduce the exposure of animals to *Fasciola* infection.

## Introduction

Fasciolosis is a disease caused by digenean trematodes of the genus *Fasciola* (Meerkhan, Razak & Younis 2013). There are two species causing infection, *Fasciola hepatica* and *Fasciola gigantica* (Avcioglu et al. 2014). *Fasciola hepatica* has a worldwide distribution but predominates in temperate zones while *F. gigantica* is found primarily in tropical regions (Abebe et al. 2010), but the two parasites overlap in subtropical regions (Khanjari et al. 2014). The primary definitive hosts of *Fasciola* parasites are the domestic ruminants (Khanjari et al. 2014), while humans become accidentally infected (Nguyen et al. 2018). Fasciolosis has been identified as a neglected tropical disease where over 180 million people in 75 countries are at risk of being infected (Liu et al. 2014; Maciel et al. 2018). Globally, over 600 million animals are at risk of getting infected with *Fasciola* parasites (Maciel et al. 2018; Rehman et al. 2016). Fasciolosis causes massive economic burden in the livestock enterprise as a result of decreased animal productivity, characterised by reduced growth rate, low quality meat and milk, infertility and diseases (Rehman et al. 2016; Shahzad et al. 2012). Prevalence of 30% – 90% of *Fasciola* infection in ruminants has been documented in Africa region (Alemneh & Ayelign 2017).

The domestic ruminants and humans get infected by ingesting of water, food and watercress or vegetation contaminated with metacercariae infective stages of *Fasciola* parasite (Mas-Coma, Valero & Bargues 2009). The biology of the parasite depends on the presence of freshwater snail intermediate hosts of family Lymnaeidae (Khanjari et al. 2014).

The snail intermediate hosts involved in the transmission of *F. gigantica* and *F. hepatica* are *Lymnaea natalensis* and *Lymnaea truncatula*, respectively (Legesse et al. 2017). The presence of suitable environmental conditions including temperature, rainfall and soil moisture influences the breeding and development of the snail intermediate hosts of *Fasciola* species (Khanjari et al. 2014). In addition, fasciolosis transmission can be influenced by climatic factors (Relf et al. 2011). The areas conducive for the breeding of the intermediate snail host include flooded pastures, grazing areas along lakeshores, slow flowing streams and river banks.

The diagnosis of fasciolosis in animals relies on the parasitological examination of ova of *Fasciola* in the faecal sample (Shahzad et al. 2012). Coprological analysis is crucial because it provides important information that helps to identify the number of infected animals releasing *Fasciola* eggs resulting in the contamination of the environment (Kleiman et al. 2005). No other diagnostic methods can provide the valuable information that coprological methods give. Thus, these are very important for epidemiological surveys.

In Kenya, there is no documented data on human fasciolosis, and the data on domestic animal fasciolosis is limited (Kanyari, Kagira & Mhoma 2009; Kithuka et al. 2002; Mungube et al. 2012). This study aimed to investigate the prevalence of fasciolosis in humans and domestic animals in Narok, Baringo and Kisumu Counties.

## Materials and methods

### Study area

**FIGURE 1:**
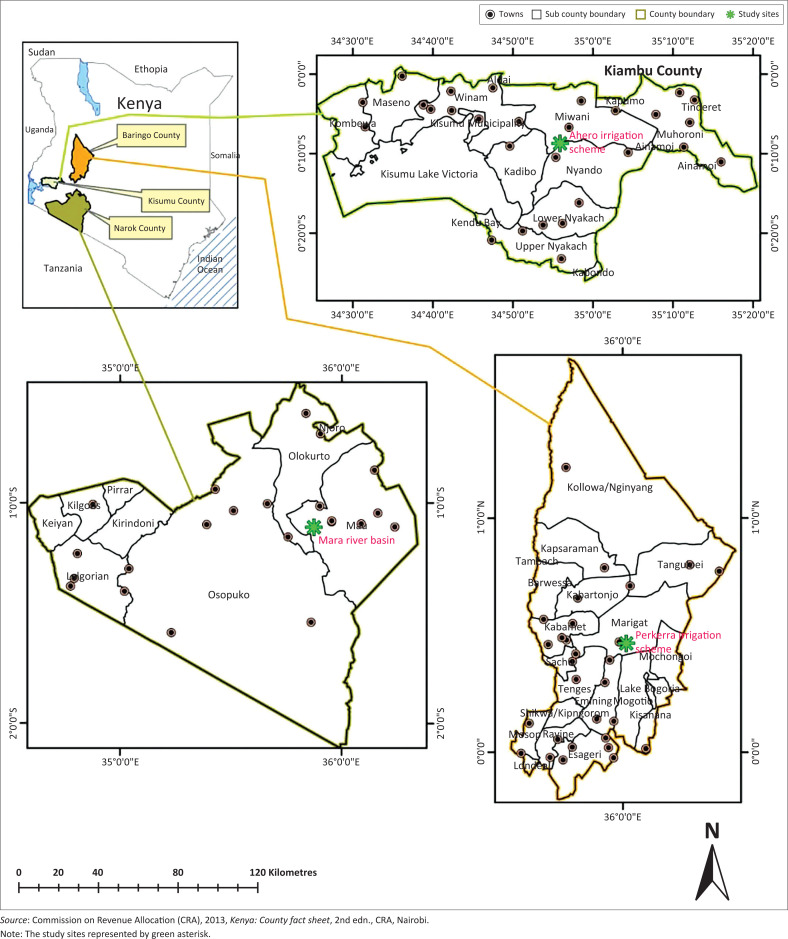
Map of Kenya showing the location of Narok, Ahero and Baringo Counties, where the samples were collected.

This study was carried out in Mara river basin in Narok County, Perkera irrigation scheme in Baringo County and Ahero irrigation scheme in Kisumu County, Kenya (Figure 1). The residents of the study area practise irrigation and livestock farming for both beef and milk production. Perkera irrigation scheme covers a total area of 2350 ha. The rainfall varies from 1000 mm to 1500 mm in the highlands to 600 mm per annum in the lowlands. The temperatures vary between 25 °C and 30 °C; however in January the temperatures rises up to 35 °C on average. The Ahero irrigation scheme has an area of 2085.9 km^2^ and annual relief rainfall between 1200 mm and 1300 mm with a mean annual temperature of 23 °C with a range of between 20 °C and 35 °C. The 13 750 km^2^ drainage area of the Mara River basin covers the agricultural and forested areas in the upper basin, the open pastureland in the middle portion of the basin and the Masai Mara Game Reserve in Kenya (1718 km^2^, all of which is within the Mara River Basin). The rainfall is bimodal and the highest annual rainfall amount is received in the high altitude areas with 1100 mm on the average.

### Sample collection

#### Stool sample collection in humans

Human stool samples were collected from Ahero Sub-county Hospital and Marigat Sub-county Hospital from January 2016 to March 2017. The participants were informed about the study and consent was obtained before inclusion in the study. A total of 272 human stool samples were collected and microscopically examined in this study. The participants were issued with clean wide-mouth polypots to collect about 10 g of stool specimen in health facility and preserved in 10% formalin solution and stored at 4 ºC – 8 ºC in cool box and transported to the laboratory for processing.

#### Stool sample collection from animals

Structured questionnaires were administered to the livestock farmers to get information on the age, sex and species of the domestic animals. A maximum of five of each of cattle, sheep and goats per herdsman were sampled. In this study, faecal samples were collected and examined from a total of 582 animals. Faecal samples were obtained directly from the rectum in disposable plastic gloves or from the ground if it was seen being dropped by the animal. The glove was turned inside out, carefully tied, labelled and transported in a cool box to the laboratory where it was processed using sedimentation technique.

#### Faecal examination by sedimentation technique

The sedimentation technique was used to find out the presence of *Fasciola* species eggs (Usip et al. 2014). Approximately 3 g of faeces was mixed with 1 L of water and 8.5 g of salt and passed through 80 µm-mesh sieve. The sieve and glass were washed at each stage. The filtrates were placed in centrifuge conical tube and centrifuged for 10 min at 5000 revolutions per minute (rpm). The supernatant was discarded. A small quantity of the sediment left at the bottom was scooped with spatula and placed on a clean slide. The prepared slide was examined under the light microscope using ×10 objective of the microscope. One drop of 1% methylene blue was added to the preparation to make the eggs more visible.

### Data analysis

Data that was collected from structured questionnaires and faecal examination was entered into a Microsoft Excel spreadsheet, thoroughly checked for errors and properly coded before subjecting to statistical analysis. The data was imported from the Microsoft Excel and analysed using Statistical Package for the Social Sciences (SPSS) version 26.0 statistical software. Descriptive statistics were used to quantify the prevalence of *Fasciola* species infection. The total prevalence was calculated by dividing the number of *Fasciola* infected animals by the total number of animals examined and then multiplied by 100.


$$\text{Total prevalence}=\frac{\text{Total number of infected animals}}{\text{Total number of animals examined}}\times 100$$ [Eqn 1]

Categorical data was analysed using univariable logistic regression analysis to determine the association of the risk factors with infection, including age, sex, species, season, and origin. A 5% significance level was used to determine where there was significant differences in the parameters.

### Ethical considerations

This study was approved by the Egerton University Research and Ethical Committee (EU/RE/DVC/009) and the Kenya National Commission for Science, Technology and Innovation (NACOSTI/P/15/8095/6943). The authors assert that all procedures contributing to this work comply with the ethical standards of the relevant national and institutional committees on human experimentation and with the Helsinki Declaration 1975, as revised in 2008 (EU/RE/DVC/009).

## Results

In this study, the human stool samples that were collected and processed using sedimentation method were all negative for *Fasciola* parasite eggs. The overall animal prevalence of *Fasciola* parasites in the three study areas was 30.9% (Table 1). A total of 582 animal faecal samples of cattle (46.0%), sheep (29.9%) and goats (24.1%) were examined in this study.

**TABLE 1:** Overall prevalence and infection status of fasciolosis with different variables.

Variable	Category	Infection status					
		Non-infected		Infected		Total	
		*n*	%	*n*	%	*n*	%
Origin	Perkera	149	25.6	72	12.4	221	38.0
	Ahero	129	22.2	75	12.9	204	35.1
	Narok	124	21.3	33	5.7	157	27.0
Total		402	69.1	180	30.9	582	100.0
Domestic animal	Cattle	178	30.6	90	15.5	268	46.0
	Goat	112	19.2	28	4.8	140	24.1
	Sheep	112	19.2	62	10.7	174	29.9
Total		402	69.1	180	30.9	582	100.0
Sex	Male	154	26.5	68	11.7	222	38.1
	Female	248	42.6	112	19.2	360	61.9
Total		402	69.1	180	30.9	582	100.0
Age	< 1 year	31	5.3	52	8.9	83	14.3
	1–4 years	291	50.0	88	15.1	379	65.1
	> 5 years	80	13.7	40	6.9	120	20.6
Total		402	69.1	180	30.9	582	100.0
Body condition	Poor	64	11.0	95	16.3	159	27.3
	Medium	175	30.1	59	10.1	234	40.2
	Good	163	28.0	26	4.5	189	32.5
Total		402	69.1	180	30.9	582	100.0
Season	Dry season	163	28.0	52	8.9	215	36.9
	Wet season	239	41.1	128	22.0	367	63.1
Total		402	69.1	180	30.9	582	100.0

*Fasciola* infection was almost similar in Perkera (12.4%) and Ahero (12.9%) irrigation schemes as compared to the infection in Narok (5.7%). The general *Fasciola* infection among cattle, sheep and goats were 15.5%, 10.7%, and 4.8%, respectively.

According to each study area, the prevalence of *Fasciola* parasite infection was found to be Perkera (32.6%), Ahero (36.8%) and Narok (21.0%). The prevalence of fasciolosis was not significantly different for the origin of the animals (*p* > 0.05) (Table 2). However, the risk of *Fasciola* infection was found to be high in the animals from Perkera and Ahero compared to the animals from Narok. The animals from Perkera and Ahero were almost twice (odds ratio [OR] = 1.771 and OR = 1.846) more likely to be infected with *Fasciola* parasites than in Narok.

**TABLE 2:** Univariate logistic regression analysis of fasciolosis with associated risk factors.

Variable	Category	Total examined	Total infected		OR	CI 95%	*p*
			*n*	%			
Origin							0.074
	Perkera	221	72	32.6	1.771	1.004–3.124	0.048
	Ahero	204	75	36.8	1.846	1.045–1.045	0.035
	Narok	157	33	21.0	-		-
Domestic animal							0.002
	Cattle	268	90	33.6	1.231	0.741–2.045	0.423
	Goat	140	28	20.0	0.393	0.207–0.749	0.004
	Sheep	174	62	35.6	-		-
Sex	Male	222	68	30.6	0.854	0.550–1.325	0.482
	Female	360	112	31.1	-		-
Age							0.000
	< 1 year	83	52	62.7	21.736	9.481–49.831	0.000
	1–4 years	379	88	23.2	2.660	1.426–4.963	0.002
	> 5 years	120	40	33.3	-		-
Body condition							0.000
	Poor	159	95	59.7	20.631	10.612–40.107	0.000
	Medium	234	59	25.2	2.496	1.419–4.389	0.001
	Good	189	26	13.8	-		-
Season	Dry season	215	52	24.2	0.675	0.427–0.427	0.094
	Wet season	367	128	34.9	-		-

OR, odds ratio; CI, confidence interval.

The results obtained show that the prevalence of *Fasciola* was significantly different between the species of the domestic animals. The prevalence was high in sheep (35.6%), followed by cattle (33.6%) and goats (20.0%). The difference of *Fasciola* prevalence in relation to animal sex was not statistically significant, although female animals had higher infection than in male animals. Evaluating the relationship between prevalence and age, it was found that the prevalence of *Fasciola* was significantly different. The prevalence was higher in the animals of less than 1 year old at 62.7% and significantly lower in those which were between 1 and 4 years old at 23.2%. The risk of infection in the animals of less than 1 year old was found to be 22 times (OR = 21.736) more than those above 5 years old.

*Fasciola* prevalence was significantly different in relation to body condition; the prevalence was higher in the animals with poor body condition (59.7%). The animals with poor body condition were almost 21 times (OR = 20.631) more likely to be infected with *Fasciola* parasites than those with good body condition. The difference between the prevalence of *Fasciola* infection and season was not significant, but there was high prevalence during wet season (34.9%) than dry season (24.2%).

## Discussion

The stool samples collected from humans were all negative for *Fasciola* eggs. This shows that the prevalence of animal fasciolosis is not predictive of the prevalence of human *Fasciola* infection in endemic regions (Mas-Coma 2004; Tolan 2011). Although coprological examination is the standard diagnosis method at clinical level (Rinaldi et al. 2012), the negative results from human faecal examination could be attributed to the shortcoming of the coprological examination which has low sensitivity because *Fasciola* takes about 10–14 weeks to release eggs (Rabia, Sabry & Nagy 2010; Rehman et al. 2016). In addition, *Fasciola* eggs are not found in the faeces because of intermittent shedding of eggs by the adult flukes in the liver (Shazad et al. 2012), presence of *Fasciola* parasites in low numbers (Rehman et al. 2016), or the flukes are still undergoing maturation (Rabia et al. 2010). To overcome the limitation of faecal examination, sensitive diagnostic methods like enzyme-linked immunosorbent assays (ELISA) can be used to test for the presence of antibodies against *Fasciola* in serum (Rehman et al. 2016), and *Fasciola* antigens faeces (Tolan 2011). Molecular techniques like polymaerase chain reaction (PCR) have also been developed which are highly specific and have been utilised in the diagnosis and differentiating the species of *Fasciola* parasites in faecal samples (Shazad et al. 2012).

The overall prevalence of *Fasciola* infection in the three study areas was 30.9%. These results are in agreement that fasciolosis is considered the most important infection in tropical regions with a prevalence of 30% – 90% in Africa (Alemneh & Ayelign 2017), and 34% prevalence of fasciolosis reported in central Kenya (Waruiru et al. 2000). The prevalence of fasciolosis reported in other regions of the world shows that in America, sheep (8.87% – 100.00%) and goat (24.50% – 100.00%), Asia, cattle (0.71% – 69.20%) and goat (0.00% – 47.00%), in Africa sheep (0.19% – 73.70%), goat (0.28% – 58.40%) and cattle (1.20% – 91.00%) and in Pakistan, sheep (14.67% – 39.20%), goat (4.08% – 28.75%) and cattle (25.46%) (Khademvatan et al. 2019).

In this study, slightly high prevalence of fasciolosis was recorded in Ahero and Perkera compared to Narok. There was no statistically significant difference (*p* > 0.05) between the prevalence of fasciolosis and the origin of the animals. The variation in the prevalence of fasciolosis in the study sites probably associated with the presence of different environmental conditions (rainfall, water logged marshy areas) and difference in climato-ecological conditions which favours the existence and development of intermediate host snails and parasite (Alemneh & Ayelign 2017; Aragaw & Tilahun 2019; Belete 2017; Khanjari et al. 2014). The high prevalence in Perkera and Ahero irrigation schemes compared to Narok, could be attributed to the irrigation activities taking place favouring the breeding of intermediate host snails. The animals from Perkera and Ahero were almost two times (OR = 1.771 and OR = 1.846) more likely to be infected with *Fasciola* parasites than in Narok.

The prevalence of fasciolosis was significantly different in relation to the species of the domestic animals. Higher prevalence was recorded in sheep, whereas goats had lower prevalence. This is in line with the results obtained from Iran (Khanjari et al. 2014). The results are also almost similar to the reports from the studies conducted in Kenya, where in Taveta, cattle, goats, sheep had prevalence of 25.9%, 23.4%, 33.3%, respectively (Mungube et al. 2012). Also, in Nyanza region, sheep and goats had prevalence of (37%) (36%), respectively, (Kanyari et al. 2009). The high prevalence of *Fasciola* infection in sheep may be attributed to their grazing habits mostly on vegetation on the ground where metacercaria are mostly found which increases their susceptibility compared to goats and cattle (Theodoropoulos 2011). In this study, the high prevalence of fasciolosis in cattle could be because *Fasciola* parasites have adapted well to bovine species (Khademvatan et al. 2019).

The difference of *Fasciola* prevalence in relation to animal sex was not statistically significant, although female animals had slightly higher *Fasciola* infection than in male animals. This is in agreement with the reports from other studies (Legesse et al. 2017; Solomon & Abebe 2007). The high prevalence of parasitism in females is attributed to pregnancy and peri-parturient period that lead to stress and reduced immune status (Khan et al. 2010). In this study, the animals (cattle, sheep and goats) examined were not separated during grazing. Therefore, both male and female domestic animals had equal exposures to contaminated grass (Tilahun et al. 2014).

*Fasciola* prevalence in relation to age was significantly different, where animals of one year old and below recorded higher prevalence and animals between one and four years old had significantly lower prevalence. This is in line with the reports from Ethiopia (Asmare & Samuel 2015; Solomon & Abebe 2007). The animals which are less than one year old were almost 22 times (OR = 21.736) more likely to be infected with *Fasciola* parasites than those above five years old. The high prevalence rate of fasciolosis in young animals can be as a result of undeveloped immunity compared to the adult animals which have developed a certain level of immunity because of repeated exposure to *Fasciola* infection (Belete 2017).

*Fasciola* prevalence was significantly higher in animals with poor body condition. This finding agrees with the results obtained from another study (Belete 2017). The animals which had poor body condition were almost 21 times (OR = 20.631) more likely to be infected with *Fasciola* parasites than those animals with good body condition. Animals with poor body condition have less resistance to infections and are therefore more susceptible to parasitic diseases compared to healthy animals.

The variation in the prevalence of *Fasciola* infection in relation to season was not significantly different, although there was high prevalence during wet season than dry season. This could be as a result of irrigation activities taking place especially in Perkera and Ahero irrigation schemes. In these areas, there is constant water for farming in the irrigation canals and also paddy fields which provide favourable breeding sites for the intermediate host snail. In areas that depend on rainfall, seasonality of fasciolosis is closely linked to the effects of rainfall and temperature, which may directly affect both intermediate hosts and the parasite (Aleixo et al. 2015). The difference in management system can contribute to the difference in the prevalence of fasciolosis regardless of the season (Legesse et al. 2017).

## Conclusion

The prevalence of fasciolosis was high in two study areas, namely, Ahero and Perkera irrigation schemes. This is as a result of the presence of marshy areas favouring the breeding of intermediate host snail and also grazing of animals along the irrigation canals and paddy fields where metacercariae of *Fasciola* parasites could be present on the vegetation. Although there was no human fasciolosis, it can pose public health risk to the residents in those areas because fasciolosis is zoonotic. Therefore, it is important to introduce measures which would assist to reduce the exposure of animals to the liver flukes, such as grazing animals away from high-risk areas, and strategic use of anthelmintics effective against liver fluke infections. In addition, an integrated approach with a combination of public health education, chemotherapy and intermediate host control could help in controlling the spread of fasciolosis.
